# Magnetic and Highly Luminescent Heterostructures of Gd^3+^/ZnO Conjugated to GCIS/ZnS Quantum Dots for Multimodal Imaging

**DOI:** 10.3390/nano11071817

**Published:** 2021-07-13

**Authors:** Bruna Lallo da Silva, Laurent Lemaire, Jean-Pierre Benoit, Fernanda Hediger Borges, Rogéria Rocha Gonçalves, Camila Fernanda Amantino, Fernando Lucas Primo, Leila Aparecida Chiavacci

**Affiliations:** 1 Department of Drugs and Medicines, School of Pharmaceutical Sciences, São Paulo State University (UNESP), Highway Araraquara-Jaú, Araraquara 14800-903, SP, Brazil; bruna.lallo@unesp.br (B.L.d.S.); camila.amantino@unesp.br (C.F.A.); fernando.primo@unesp.br (F.L.P.); 2 MINT, INSERM, CNRS, SFR-ICAT, UNIV Angers, 49000 Angers, France; laurent.lemaire@univ-angers.fr (L.L.); jean-pierre.benoit@univ-angers.fr (J.-P.B.); 3 PRISM, SFR-ICAT, UNIV Angers, 49000 Angers, France; 4 Laboratório de Materiais Luminescentes Micro e Nanoestruturados—Mater Lumen, Departamento de Química, FFCLRP, Universidade de São Paulo (USP), Ribeirão Preto 14040-901, SP, Brazil; fernanda.hediger.borges@usp.br (F.H.B.); rrgoncalves@ffclrp.usp.br (R.R.G.)

**Keywords:** quantum dots, magnetic resonance imaging, luminescent properties, cytotoxicity

## Abstract

In recent years, the use of quantum dots (Qdots) to obtain biological images has attracted attention due to their excellent luminescent properties and the possibility of their association with contrast agents for magnetic resonance imaging (MRI). In this study, Gd^3+^/ZnO (ZnOGd) were conjugated with Qdots composed of a gadolinium-copper-indium-sulphur core covered with a ZnS shell (GCIS/ZnS Qdots). This conjugation is an innovation that has not yet been described in the literature, and which aims to improve Qdot photoluminescent properties. Structural and morphological Qdots features were obtained by transmission electron microscopy (TEM), Fourier transform infrared spectroscopy (FTIR), X-ray diffraction (XRD) and thermogravimetric analyses (TGA). The photoluminescent properties were examined by emission (PL) and excitation (PLE) spectra. A new ZnOGd and GCIS/ZnS (ZnOGd-GCIS/ZnS) nanomaterial was synthesized with tunable optical properties depending on the ratio between the two native Qdots. A hydrophilic or lipophilic coating, using 3-glycidyloxypropyltrimethoxysilane (GPTMS) or hexadecyltrimethoxysilane (HTMS) on the surface of ZnOGd-GCIS/ZnS Qdots, was carried out before assessing their efficiency as magnetic resonance contrast agents. ZnOGd-GCIS/ZnS had excellent luminescence and MRI properties. The new Qdots developed ZnOGd-GCIS/ZnS, mostly constituted of ZnOGd (75%), which had less cytotoxicity when compared to ZnOGd, as well as greater cellular uptake.

## 1. Introduction

Quantum dots (Qdots), in addition to being resistant to photobleaching, have a long luminescence lifetime compared to organic fluorophores. As a consequence, they have been used to obtain optical images in diagnosis [1,2,3]. Among the new generations of Qdots, ZnO Qdots are promising materials for imaging, especially due to their low toxicity, their excellent luminescent properties and their inexpensive production [4,5]. Moreover, ZnO Qdots can be functionalized by using the hydroxyl groups on their surface with small organic molecules such as organosilanes [6,7,8] or polymers such as chitosan [9]. Nevertheless, the main limitation regarding the use of ZnO Qdots is their excitation and emission features. Indeed, their excitation range is usually in the ultraviolet (UV) region, which can be strongly deleterious for cell DNA and, therefore, jeopardize its in vivo use. Moreover, such irradiation can also lead to biological sample autofluorescence due to the presence of natural molecules that prevent the detection of Qdot emission [10,11]. Finally, their photoluminescence intensity decreases as the UV irradiation exposure time increases, which also leads to a significant change in luminescence color related to the particle’s aggregation [12].

To overcome these instability issues, surface modification with organosilanes [6,8,12,13,14] and ionic liquids [13], or association with nanocarriers [9], has been proposed. However, despite improvements in the emission features using magnesium [15] or lanthanides [16] as doping agents, UV excitation remains a limitation for ZnO Qdot use for biological purposes. However, developments in solar cell research have come with interesting results that can be of interest to biology. It has been shown that, after creating heterostructures between ZnO nanoparticles and other Qdots such as CuInS_2_ (CIS) [17] or CIS/ZnS Qdots [18], a shift of absorption in the visible region can be observed.

Based on this research, we propose to elaborate a new class of Qdots in which the heterostructures will be doped with gadolinium. Thus, Qdots can be efficiently used as a luminescent agent through the optimization of luminescent properties, allowing a wide excitation range (UV and visible) and its use as a magnetic agent for both imaging techniques. Hence, both functionalities, integrated into a single particle, create a very powerful platform that can be used in highly complementary imaging techniques (fluorescence imaging and magnetic resonance imaging-MRI) for biological media. In order to do that, gadolinium-doped ZnO Qdots (ZnOGd) were associated with Qdots based on gadolinium, copper, indium, and sulphur/zinc sulphide (GCIS/ZnS) by heat treatment. To reveal the contrast properties in the magnetic resonance imaging, ZnOGd-GCIS/ZnS Qdots powders were coated with either 3-glycidyloxypropyltrimethoxysilane (GPTMS) or hexadecyltrimethoxysilane (HTMS) before being dispersed in water or in a lipidic phase, respectively, as encountered in lipid nanocapsule (LNC) formulation.

## 2. Materials and Methods

### 2.1. Materials

1-Dodecanethiol (DDT), gadolinium (III) 2,4-pentanedionate hydrate, copper (II) acetylacetonate, indium (III) 2,4-pentanedionate, zinc acetate dihydrate, lithium hydroxide, 3-glycidyloxypropyltrimethoxysilane (GPTMS) and hexadecyltrimethoxysilane (HTMS), were purchased from Sigma Aldrich, USA. Absolute ethanol and heptane were purchased from Synth-Brazil. Lipophilic Labrafac^®^ WL 1349 (caprylic-capric acid triglycerides; European Pharmacopeia, IVth, 2002) was purchased from Gattefossé S.A. (Saint-Priest, France). Lipoïd S75-3 (soybean lecithin at 69% of phosphatidylcholine) and Kolliphor^®^ HS 15 (a mixture of free polyethylene glycol 660 and polyethylene glycol 660 hydroxystearate) were provided by Lipoïd GmbH (Ludwigshafen, Germany) and BASF (Ludwigshafen, Germany), respectively. NaCl was purchased from Prolabo (Fontenay-sous-Bois, France). Filtered and deionized water was obtained from a Milli-Q plus^®^ system (Millipore, Burlington, MA, USA).

### 2.2. Sample Preparation

#### 2.2.1. Gd^3+^/ZnO Quantum Dot (Qdot) Synthesis

ZnOGd Qdots were obtained by a sol-gel method proposed by Spanhel and Anderson [19]. First, the Zn_4_O(Ac)_6_ tetrameric precursor was prepared through refluxing of an ethanolic solution containing 0.05 M zinc acetate at 80 °C for 2 h. ZnO Qdot colloidal suspensions were obtained from the precursor by hydrolysis and condensation reactions with the addition of LiOH in a hydrolysis ratio, r = ([OH]/[Zn]), of 1.0 at 60 °C for 1 h in an ultrasound bath. ZnO Qdots were doped with Gd by adding a gadolinium chloride ethanol solution to a Zn_4_O(Ac)_6_ tetrameric precursor. The Gd concentration was 20 mol % in relation to the Zn mol %. LiOH was added in a hydrolysis ratio, r = [OH]/[Zn + Gd] of 1.0 at 60 °C for 1 h in an ultrasound bath. Powders were extracted from the colloidal suspensions according to the method described by Meulenkamp [20], using heptane as a non-solvent. This yielded a white precipitate that was centrifuged for 10 min at 4000 rpm (3220 g) and dried at 35 °C for 24 h.

#### 2.2.2. Gadolinium, Copper, Indium, and Sulphur/Zinc Sulphide (GCIS/ZnS) Qdots

GCIS/ZnS Qdots were synthesized according to the method of Yang et al. [21], with some modifications [3]. 1-Dodecanethiol (DDT) was used as a surface ligand, sulphur source, and reaction solvent. First, gadolinium (III) 2,4-pentanedionate hydrate (0.1 mmol), Copper (II) acetylacetonate (0.2 mmol) and indium (III) 2,4-pentanedionate (0.2 mmol) in different proportions were mixed in 15 mL of DDT under an argon atmosphere. The mixture was degassed at 80 °C for 30 min. The temperature was then increased to 245 °C for 2 h. The heat source was removed and one proportion of 4 mL sample of the core GCIS was removed and purified with an excess of ethanol. For the ZnS shell, the mixture was heated at 220 °C and a solution containing 110 mg of zinc acetate, 8 mL of 1-octadecene, and 1 mL of oleic acid was added (one amount of solution split into four proportions was added over a 20-min interval). Finally, ethanol was added, and the suspension was centrifuged at 4000 rpm (3220 g) for 15 min (this procedure was repeated 4 times).

#### 2.2.3. Heat Treatment to Promote Qdot Junctions

ZnOGd Qdots were coupled with GCIS/ZnS Qdots following the procedure described by Donat et al. [18], with some modifications. Qdot powders were mixed in trichloromethane and magnetically stirred at room temperature until complete evaporation of the solvent. Different proportions between ZnO, ZnOGd and GCIS/ZnS Qdots were obtained (Table 1). Then, heat treatment was carried out at 260 °C for 5 min to promote the junction between ZnOGd Qdots to GCIS/ZnS Qdots.

#### 2.2.4. Qdot Surface Modification

For the hydrophilic Qdot modifications, 10 mg of ZnO-GCIS/ZnS were dispersed in 12 mL of ethanol. The surface modifier GPTMS (0.1 mol/L) was added into the Qdot dispersion. 0.05 mol/L of LiOH was added to the mixture and the reaction was carried out in an ultrasound bath for 30 min. The precipitate formed was centrifuged and the powder was dispersed in water (5 mg/mL). For the hydrophobic Qdot modifications, 10 mg of ZnO-GCIS/ZnS powder were dispersed in 12 mL of ethanol. The surface modifier, HTMS (0.045 mol/L), was added into the Qdot dispersions. We added 0.03 M of LiOH to the mixture and the reaction was carried out in an ultrasound bath for 15 min at 35 °C. The precipitate formed was centrifuged, dried and the powder was dispersed in Labrafac^®^ (25 mg Qdots in 1000 mg of labrafac^®^).

#### 2.2.5. Qdot Association with Lipid Nanocapsules

The preparation of the lipid nanocapsules (LNC) was performed through the phase inversion method [22]. Briefly, an oil/water (O/W) emulsion was prepared. Firstly, 25.7 mg of HTMS-coated Qdots were dispersed in Labrafac^®^ (1028 mg). Then, Koliphor (846 mg), Lipoid S75-3 (75 mg), NaCl (148 mg) and Milli-Q water (2970 mg) were added. Three temperature cycles, between 40 and 95 °C, were then carried out, followed by rapid cooling after the last cycle using an excess of cold water (2000 mg).

### 2.3. Characterization

#### 2.3.1. Evaluation of Qdot Luminescent Properties

Qdots photoluminescent properties were evaluated by measuring the emission (PL) and excitation spectra (PLE). Spectra were recorded in the visible range on a spectrometer Fluorolog 3 Horiba Scientific FL3-22 at room temperature, equipped with a double excitation monochromator, a double emission monochromator, and a Hamamatsu R928 photomultiplier with a Xe lamp (450 W). For PL measurements, each sample was excited at the optimal wavelength, defined by the PLE spectrum. Images were also collected after excitation of the Qdots using either an ultraviolet (UV) lamp with a fixed excitation wavelength (365 nm) or a Maestro in vivo imaging system with different wavelengths (455, 523, 595, 605 and 635 nm).

#### 2.3.2. Fourier Transform Infrared Spectroscopy (FTIR)

Absorption spectra in the infrared region (4000–400 cm^−1^) were obtained using a Vertex 70.

#### 2.3.3. Transmission Electron Microscopy (TEM)

Transmission electron microscopy (TEM) images were obtained with a Philips Electronic Transmission Microscope (model CM 200; SUPER TWIN) operated at 200 kV. The diluted Qdots dispersed in ethanol were deposited on carbon grids and kept overnight. Image analysis was carried out with the ImageJ software (National Institutes of Health, Bethesda, MD, USA).

#### 2.3.4. X-ray Diffraction (XRD)

The X-ray diffraction (XRD) analyses were performed on a Siemens D5000 diffractometer using Cu-Kα radiation with a wavelength, λ, equal to 1.5418 Å. The diffraction intensity was measured in the 2θ range of 5–90° with an increment of 0.005°, and 1 s per step.

#### 2.3.5. Thermogravimetric (TG) Analysis

TG analysis was carried out on an SDT 600 simultaneous differential thermal analysis (DTA)/TG (TA Instruments). Thermograms were obtained in the temperature range of 25 to 700 °C with a heating rate of 10 °C min^−1^ in air atmosphere.

#### 2.3.6. Magnetic Resonance Imaging of GPTMS-Qdots Dispersed in Water, and Hexadecyltrimethoxysilane (HTMS)-Qdots within an Lipid Nanocapsule (LNC) Preparation

Magnetic resonance imaging was performed using a 7T scanner (Biospec 70/20 Avance III, Bruker Wissembourg, France) equipped with BGA12S gradient system (675 mT/m). Emission and reception were ensured by a 72 mm diameter resonator. T_1_- and T_2_ weighted images were acquired using a spin-echo sequence. For T1-weighting, the repetition time was fixed to TR = 200 ms with echo time TE = 8 ms. To acquire T2-weighted images, TR = 2000 ms and TE = 104 ms were used. In both cases, images were acquired with a field of view = 6 cm × 4 cm, a matrix = 256 × 192, and a slice thickness of 2 mm.

#### 2.3.7. Cytotoxicity Assay Using Resazurin Test

Mouse embryonic fibroblast cells (NIH/3T3-ATCC^®^ CRL-1658^™^) were seeded into a 96-well plate (5 × 10^3^ cells/well). After 24 h, GPTMS coated Qdots (ZnOGd, GCIS/ZnS and ZnOGd-GCIS/ZnS (75-25) were added at a concentration ranging from 5–250 µg mL^−1^ for a 4-h incubation period. Cells were then washed and allowed to grow for a further 24 h before the addition of 20 µL of resazurin solution (25 µg mL^−1^ in phosphate-buffered saline (PBS). After 4 h, wells analyses were carried out in the microplate reader EnSpire^®^ (PerkinElmer, Waltham, MA, USA) at 570 nm and the basal absorbance was corrected at 590 nm. The percentage of viable cells was calculated following Equation (1). The analyses were performed in triplicate.

(1) $$\mathrm{Viable}\;\mathrm{cells}\;(\%)\;=\;\left(\frac{O.D.sample}{O.D.control}\right)\times \;100$$

#### 2.3.8. Cell Uptake Assay

The cell uptake assay was performed using the NIH-3T3-ATCC^®^ CRL-1658™ cell line. Cells were seeded into 24 wells of 3 × 104 cells/well target plates. Different concentrations were tested for the quantum dots. Incubation times were tested were 6 and 24 h. After the incubation time, the cells were washed in PBS to remove the quantum dots that were not captured by the cells, and the fluorescence analyzes were performed in an EnSpire^®^ microplate reader (PerkinElmer, Waltham, MA, USA) with an excitation wavelength of 360 nm and emission at 550 nm.

## 3. Results and Discussions

### 3.1. Qdot Characterization

Heat treatment (HT) has been used to induce the transformation of phases and structures to improve the properties of many materials, as well as promoting coalescence between particles. Thus, we have used this principle to promote the junction between Qdots [23,24,25]. Therefore, as shown by Donat et al., the conjugation of ZnO nanoparticles with Qdots containing copper, indium, sulphur (CIS), and zinc sulphide (ZnS), at 400 °C, is of interest for solar-driven photocatalysis [18]. However, in our study, the heat treatment carried out on the samples at similar temperatures led to a loss of luminescence and thus required an optimization of the temperature to a maximum of 260 °C.

**Figure 1:**
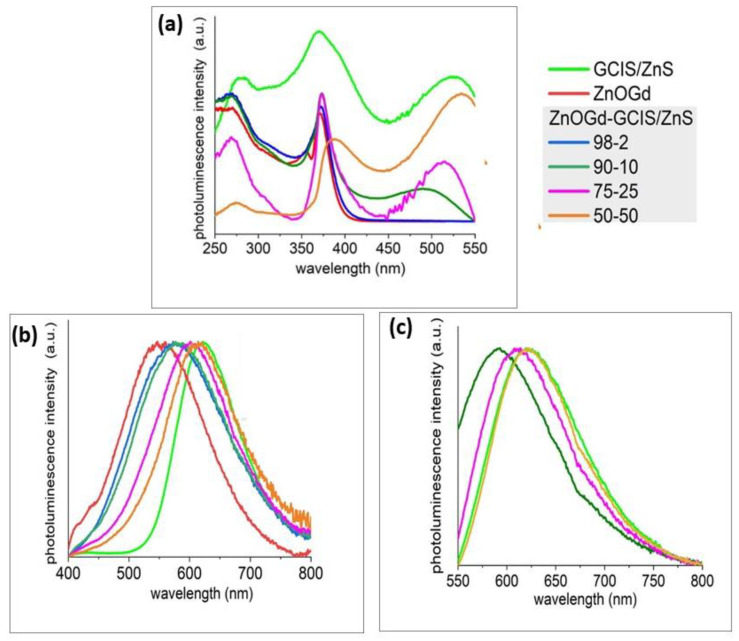
Excitation (PLE)—(**a**), and emission (PL) spectra of Qdot powders after excitation at 360 nm (**b**) and 500 nm (**c**).

Before conjugation, ZnOGd Qdots were characterized by a PLE spectrum with two bands at 260 nm and 360 nm, whereas, for GCIS/ZnS Qdots, a third band was observed at approximately 520 nm (Figure 1a). When the excitation is performed at 360 nm, the PL spectra of ZnOGd Qdots show emission at 550 nm, and for GCIS/ZnS, emission at 620 nm (Figure 1b) occurs. Interestingly, when the heat treatment is performed, the resulting material exhibits a broadband emission after excitation at 360 nm, with a maximum wavelength between the maxima of each of the pure untreated materials. It is worth highlighting that, when the GCIS/ZnS to ZnOGd ratio was increased, the redshift increased. However, the shift was not linearly correlated to the amount of GCIS/ZnS; for 25% of GCIS/ZnS a redshift of 53 nm was found while for 50% of GCIS/ZnS, the redshift was 68 nm (Table 2). This excitation ability at different wavelengths is attributed to ternary or quaternary Qdots containing copper, [26] represented in our study by GCIS/ZnS. Although interesting, the emission exhibited by major Qdots requires an excitation in the UV range, which limits their applications in a biological context due to potential tissue damage [27] and to the strong auto-fluorescence signal background of the biological medium [10,11]. One of the most relevant goals of this work was to obtain a low toxicity material based on a majority of ZnO which, when conjugated to a small amount of GCIS/ZnS, can have its photophysical properties displaced to the visible range.

The composite material obtained after heat treatment shows a significant change in fluorescence properties as the GCIS/ZnS amount increases. Indeed, it is possible to excite ZnOGd-GCIS/ZnS Qdots at the third PLE peak, located at 500 nm. Similar to Figure 1b, a redshift was also observed as the GCIS/ZnS percentage increased (Figure 1c) after excitation at 500 nm. For the ZnOGd-GCIS/ZnS prepared using 25% of GCIS/ZnS, emission at 613 nm was observed, while using 50% of GCIS/ZnS the broadband emission was located around 620 nm, which is similar to GCIS/ZnS, without ZnOGd emission. Therefore, a compromise between redshift and GCIS/ZnS amount was applied to select the optimized sample proportions. An intermediate PL was clearly observed between the ZnOGd and GCIS/ZnS Qdots, and the maximum redshift was found for ZnOGd-GCIS/ZnS Qdots prepared using the proportions of 75% ZnOGd and 25% GCIS/ZnS. Hence, the latter was selected as the best candidate to be applied for further characterization.

**Figure 2:**
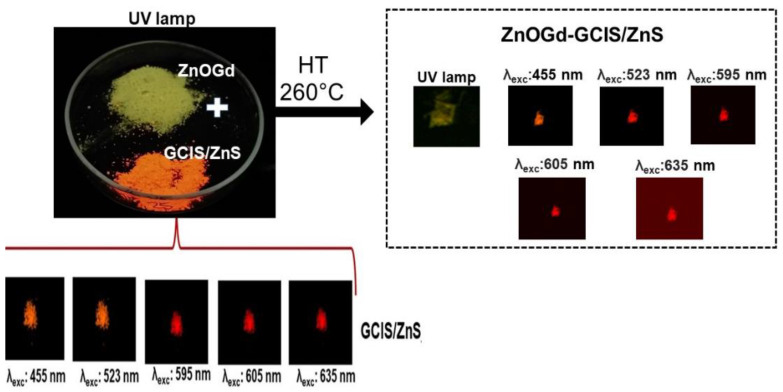
Emissions of ZnOGd under ultraviolet (UV) lamp at 254 nm and emissions of GCIS/ZnS and conjugated Qdots ZnOGd-GCIS/ZnS (75% of ZnOGd and 25% of GCIS/ZnS) using different wavelengths of excitation.

**Figure 3:**
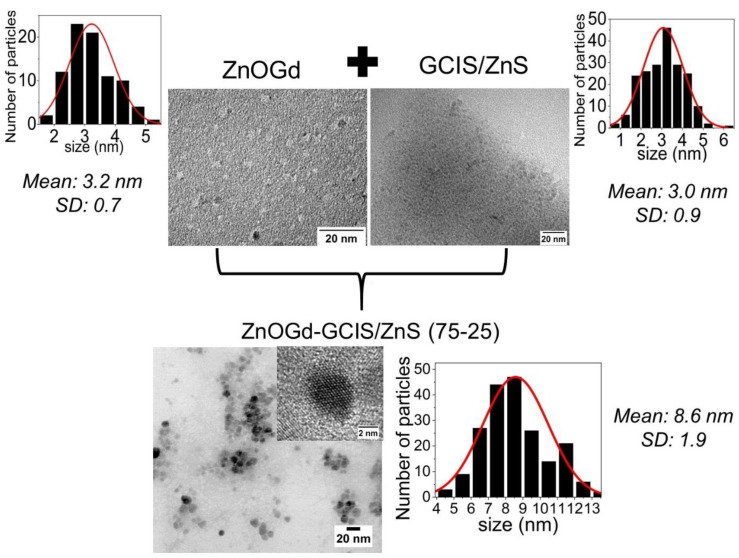
Transmission electron microscopy (TEM) images and size distributions of ZnOGd, GCIS/ZnS and the conjugated Qdots after heat treatment at 260 °C.

Figure 2 shows the emission images of the ZnOGd and GCIS/ZnS Qdots at different wavelengths (455, 523, 595, 605 and 635 nm) using a Maestro in vivo imaging system. An orange color emission was detected for the GCIS/ZnS Qdots, while the ZnO Qdots have a greenish luminescence. It was also possible to observe that the GCIS/ZnS Qdots show luminescence when excited with a UV lamp, but also, when excited outside of UV region, from blue to red (455–635 nm). However, the ZnOGd Qdots exhibited an emission only after excitation using a UV lamp. After the Qdot hetero-conjugation, promoted by the heat treatment, the Qdots could be excited in the UV region, and also range from blue to red (455–635 nm), with an orange color emission. The ability to excite these new Qdots, outside the UV, demonstrate an important limitation of ZnO for use in biological images that need to be overcome, as the autofluorescence of cells results in difficulty in ZnO Qdot detection [10,11,28]. In addition, UV excitation can damage tissues [27]. It is important to highlight that these new Qdots contain mostly ZnO, which is a material considered safe for human use, as it is biocompatible and non-toxic. Therefore, in this article we describe new Qdots containing mostly ZnO, with optimized luminescent properties, however. For size evaluation and a possible indication that the reaction between the two Qdots occurred, a TEM analysis was performed. This showed that, after the heat treatment, a mixture of ZnOGd-GCIS/ZnS (75-25) nanoparticles with an average size of 8.6 nm was produced. The initial sizes of ZnOGd and GCIS/ZnS were 3.2 nm and 3.0 nm, respectively (Figure 3).

**Figure 4:**
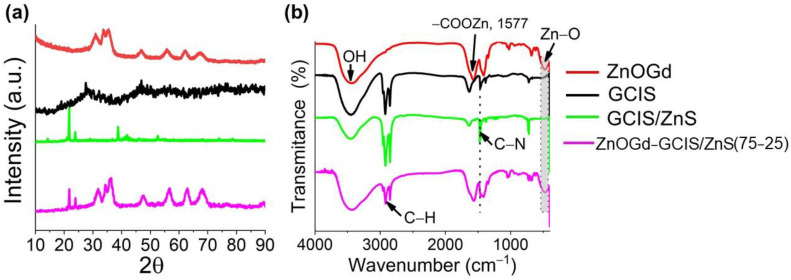
X-ray diffraction (**a**) and Fourier transform infrared spectroscopy (FTIR) (**b**) of ZnOGd, GCIS, GCIS/ZnS and the association of ZnOGd with GCIS/ZnS Qdots, 75% and 25%, respectively.

Figure 4 shows X-ray diffraction (A) and Fourier transform infrared spectroscopy (FTIR) spectra (B) of ZnOGd, GCIS, GCIS/ZnS and the ZnOGd-GCIS/ZnS.

The XRD peaks of ZnOGd Qdots correspond to the ZnO hexagonal phase with the wurtzite structure, indexed in JPDS file 36-1451. GCIS Qdots presented a diffraction pattern similar to that found by Yang et al., [3] in which three intense peaks are presented, located at 2θ with values of 27.46°, 46.87° and 56.05°. According to Donat et al. [18], these peaks correspond to the crystallographic planes (112), (204)/(220) and (116)/(312) to the tetragonal chalcopyrite structure of CIS Qdots. Therefore, GCIS Qdots show the planes corresponding to the tetragonal chalcopyrite structure. For GCIS/ZnS Qdots, new intense peaks were formed, wherein the most intense was located at 2θ, with a value of 21.82°.

Gromova at al. [29] attributed the small angle X-ray scattering (SAXS) peaks to the DDT periodic lamellar structure, formed at 100 °C, after its cooling in room temperature. Furthermore, the characteristic distance found d = 2π/q1 = 34.9 Å corresponds to a good approximation of twice the length of a DDT ligand. The possibility of forming a mixture of phases depending on the molar ratio used between In and Cu for the Qdot formation was discussed by Zhang et al. [30]. In that study, Qdots were synthesized using different molar ratios between Cu, In and Zn. With a ratio of Cu to In of 1:1, the researchers suggested the formation of the co-existence of the chalcopyrite structure of the CIS Qdots and the wurtzite structure of the CuS. The ratio used between Cu and In in our research was also 1:1; however, it is noteworthy that our synthesis has differences when compared to the article by Zhang et al., which did not show diffractogram profiles at 2θ smaller 25°. Jayabharathi et al. [31] developed copper/silver nanoclusters protected by DDT. The XRD profiles contained a series of peaks at low angles in the range of 2θ = 10–40°.

According to this data, we hypothesized that, after the introduction of ZnS, the reagents, such as 1-octadecene, oleylamine and oleic acid, interacted forming a new crystalline structure responsible for the peaks at low angles.

To confirm this hypothesis, we performed a synthesis with all reagents, excluding salts containing gadolinium, copper, indium, and zinc and subjecting all other reagents to an increase and decrease in temperature for the time stipulated in accordance with Section 2.2.2. After the end of the reaction, a white precipitate was formed. The X-ray diffraction of this precipitate showed that all peaks corresponded to those found for GCIS/ZnS. Therefore, the formation of a new phase was discarded, indicating that a new organic structure was formed through the interaction of the organic reagents. Akdas et al. [32] showed that DDT can interact with ODE at high temperatures and form dodecyloctadecylsulfide, which induces additional impurities that cause problems in the purification process. Therefore, the peaks found in the diffraction correspond to the organic structure, probably dodecyloctadecylsulfide, which even after successive purification cycles with ethanol is not fully removed. The peaks corresponding to the tetragonal chalcopyrite structure can be hidden due to the presence of intense peaks. However, it is important to highlight that there is the presence of GCIS/ZnS Qdots, as it is possible to observe their luminescence (Figure 1 and Figure 2) and morphology (Figure 3).

After the association of ZnOGd Qdots with GCIS/ZnS through heat treatment at 260 °C, the peaks of GCIS/ZnS Qdots were not found at angles higher than 2θ = 25°. However, after the Qdot conjugation, the most intense peaks of the GCIS/ZnS were present at 2θ = 21.68° and 24.11°.

In Figure 4b, the FTIR spectrum of ZnOGd Qdots contains an absorption band at 3400 cm^−1^, characteristic of OH hydroxyl groups [33]. Moreover, bands associated with acetate groups in the region of 1400–1600 cm^−1^ and characteristic vibrations of the Zn-O at 470 cm^−1^, were also observed [34], and there is a displacement of these vibrations in the conjugated Qdots. GCIS and GCIS/ZnS Qdots present symmetrical and asymmetrical stretching attributed to the C–H bond, located between 2963 and 2853 cm^−1^ [35]. This link can be associated with DDT, as well as with other reagents used to form ZnS.

Jayabharathi et al. [31] found two bands for the DDT associated with the asymmetric and symmetric stretching of methylene at 2920 and 2850 cm^−1^, 2920 and 2850 cm^−1^, respectively. GCIS/ZnS Qdots also have vibrations in these wavenumbers, with the asymmetric vibration at 2920 cm^−1^, shifting to around 2917 cm^−1^ in the conjugated Qdots, which suggests an interaction between the DDT and the conjugated Qdots.

After conjugation of the ZnOGd with GCIS/ZnS, it was possible to observe the characteristic vibrations of ZnOGd and GCIS/ZnS. A band associated with the symmetric deformation of CH_2_ was found at 1416 cm^−1^ GCIS/ZnS. After Qdot conjugation, carried out through the heat treatment, these vibrations continue to appear. This may indicate that the heat treatment does not remove the reagents used in the synthesis [36]. However, this can facilitate Qdot dispersion in organic solvents and act as stabilizing agents. DDT was found to be a stabilizing agent for Qdots [29].

**Figure 5:**
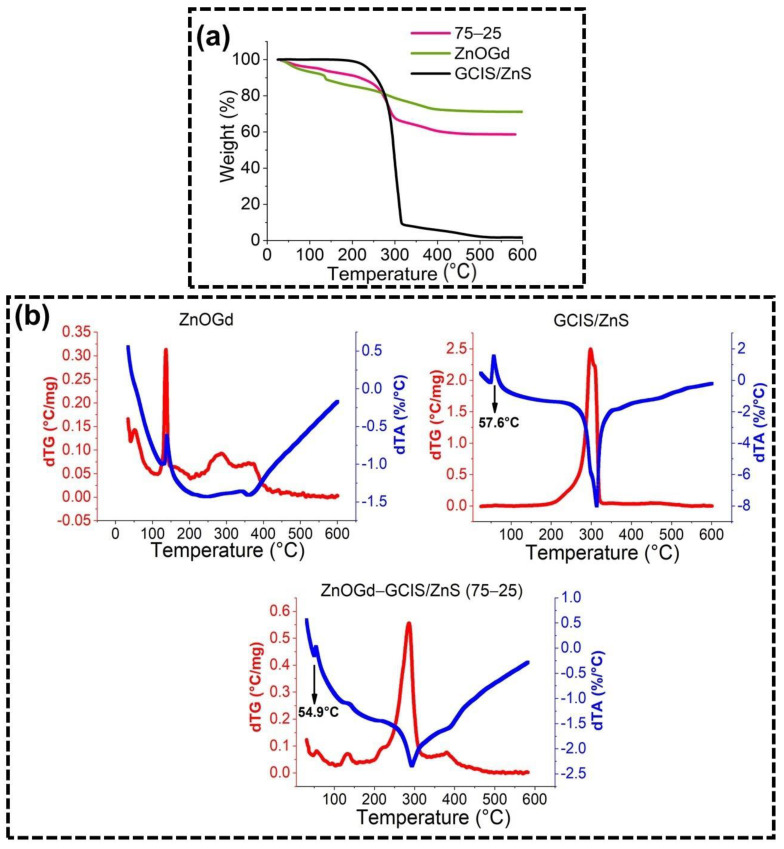
Thermogravimetric analyses of ZnOGd, GCIS/ZnS and ZnOGd-GCIS/ZnS (75-25) (**a**) and their respective DTA and DTG (**b**).

Figure 5 shows the thermogravimetric (TG) analysis of ZnOGd, GCIS/ZnS Qdots, conjugated using the proportions of 75% and 25%, respectively, after heat treatment at 260 °C. ZnOGd showed two important events. The first event, up to 100 °C, can be associated with the volatilization of solvent residues, such as ethanol and water. The second event corresponds to the loss from 100 °C (about 5% to 120 °C) up to 400 °C, and is characteristic of the residue’s decomposition of organic compounds, such as the loss of the acetate group from the zinc acetate used to perform the ZnOGd synthesis [6,24]. GCIS/ZnS Qdots have a fusion event reaching a maximum temperature at 57.16 °C. After 200 °C, the TG data showed a composition due to the organic solvents present, such as DDT. Wada et al. [37] showed a higher weight loss of CIS/ZnS in TG analysis, attributed to the DDT at high temperatures, which decreased after the Qdot surface modifications. The conjugated Qdots showed an intermediate characteristic between the mass loss of ZnOGd and GCIS/ZnS (A). However, the fusion event that was previously found at 57 °C for GCIS/ZnS shifted to 54 °C. There was a difference in the fusion rate of about 10 times, which, for GCIS/ZnS Qdots was 0.4142 °C/min, and for the conjugated Qdots, it was 0.04155 °C/min (B). This difference in both rate and fusion temperature indicates the interaction of ZnOGd with GCIS/ZnS Qdots.

For the conjugated Qdots, after heat treatment at 260 °C, the synthesis reagents were not totally eliminated, as is shown by the high loss of mass at high temperatures. This result was evidenced in the XRD and FTIR techniques and again demonstrated by thermogravimetric analysis. Thus, we suggest that DDT and other reagents used for synthesis are arranged in such a way that they interact with the Qdots and protect their luminescence and, then, cannot be eliminated completely.

### 3.2. Magnetic Resonance Images of GPTMS-Qdots Dispersed in Water and HTMS-Qdots within LNC Preparation

**Figure 6:**
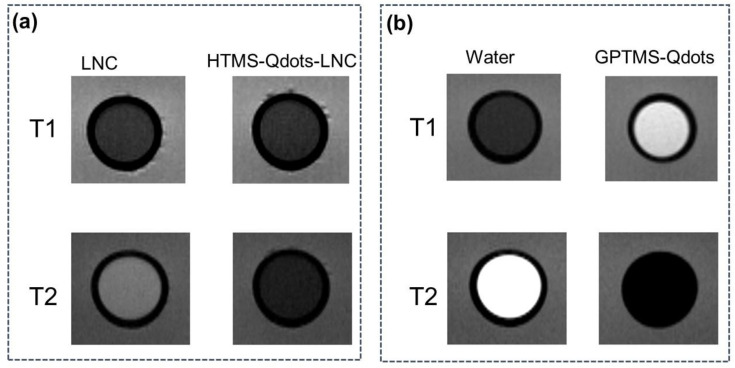
T1 and T2-weighted images of un-loaded LNC preparation and LNC loaded with HTMS-coated LNC (**a**). T1 and T2-weighted images of pure water and GPTMS-coated Qdots (**b**).

For the evaluation of the composite material magnetic properties, Qdot surface modifications were carried out to help their dispersion either in water or in an oil phase. Organosilanes were chosen for this reason, as they have low toxicity and they covalently bond to the Qdot hydroxylated surface, thereby creating a protective barrier between the Qdots and the solvents [38,39] without impairing the luminescence properties of the native ZnO Qdots [8,12,38]. This strategy was used to stabilize the composite material and allow magnetic resonance detection. To collect the images, we used the conjugated Qdots (ZnOGd-GCIS/ZnS 75-25). Figure 6 shows T1 and T2-weighted magnetic resonance images of LNC loaded with HTMS-Qdots (a) and GPTMS coated Qdots dispersed in water (b). Both preparations led to an excellent T2 contrast (observed as negative contrast—dark images in Figure 6a,b on the top), whereas only the GPTMS-Qdots directly dispersed in water led to a T1-effect (observed as positive contrast—bright images). In the latter case, the T1 effect can be explained by the thin layer of coating that does not fully isolate the Qdots from the surrounding water and, therefore, allows the longitudinal relaxation process as observed, for example, by SiO_2_-coated layered gadolinium hydroxides [40]. The T2 effect observed in both samples is classic for a particulate system that creates a local magnetic field inhomogeneity.

ZnOGd Qdots had a greater contribution to MRI contrast than GCIS/ZnO Qdots. This fact can be explained because there is a low amount of Gd in the GCIS/ZnS Qdots due to the amount of organic matter from the reaction between DDT and ODE, as was previously demonstrated with the techniques of thermogravimetric analyses, infrared spectroscopy and XRD. Consequently, this material was inefficient as a magnetic resonance contrast agent (data not shown).

### 3.3. Cytotoxicity Assay

**Figure 7:**
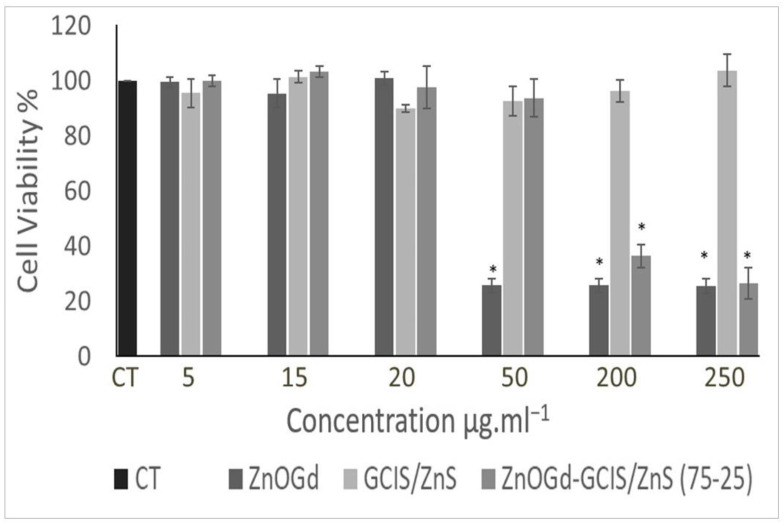
Cytotoxicity of quantum dots modified with GPTMS and dispersed in water (ZnOGd, GCIS/ZnS and ZnOGd-GCIS/ZnS (75-25)) on 3T3/NIH fibroblast cells. Cells were incubated with different concentrations (5, 15, 20, 50, 200 and 250 μg·mL^−1^) for 4 h in 5% CO_2_ and 37 °C. Data are presented as mean ± standard error of the mean (SEM) of three independent experiments. Statistical significance was determined using the one-way analysis of variance (ANOVA) test followed by the Tukey post-test for multiple comparisons. (CT = control, * *p* < 0.05).

The Qdot cytotoxicity, modified with GPTMS and dispersed in water, was evaluated at a range of concentrations on NIH/3T3 cells (Figure 7). GCIS/ZnS Qdots showed a limited impact on cell viability (>80%) in all explored concentrations, indicating that GCIS/ZnS induces low cytotoxicity to NIH/3T3 cells. For ZnOGd Qdots, no impact on cell viability was observed for concentrations up to 20 μg·mL^−1^. At higher concentrations, cell viability did not exceed 20%. After Qdot conjugation, ZnOGd-GCIS/ZnS resulted in higher cell viability when compared to ZnOGd. It is important to highlight that these Qdots comprise mostly of ZnOGd (75%). While at 50 μg·mL^−1^, cell viability of ZnOGd was about 20%; for the ZnOGd-GCIS/ZnS, the viability was about 90%, similar to GCIS/ZnS. These findings reveal that, even with small proportions of GCIS/ZnS (25%) added to ZnOGd Qdots, the resulting Qdot has less cytotoxicity when compared to ZnOGd.

### 3.4. Cell Uptake Assay

**Figure 8:**
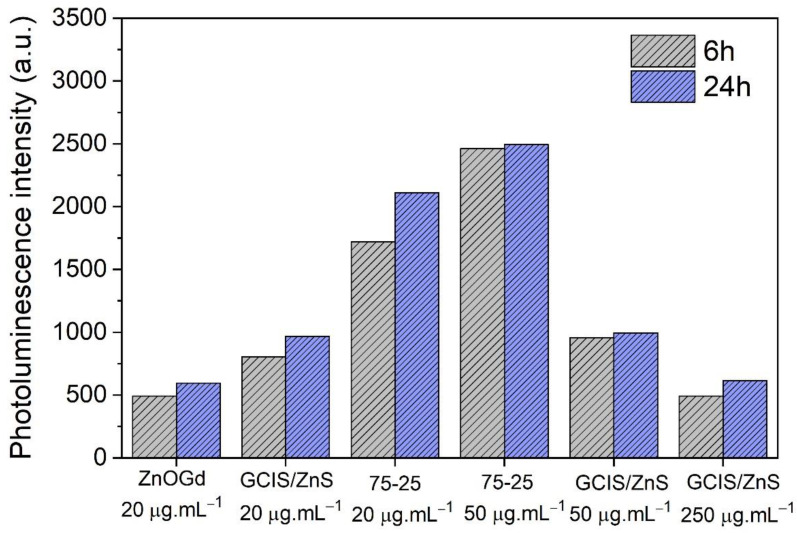
Cell uptake of quantum dots modified with GPTMS and dispersed in water (ZnOGd, GCIS/ZnS and ZnOGd-GCIS/ZnS (75-25)) on 3T3/NIH fibroblast cells. Cells were incubated with different concentrations for 6 h and 24 h. Cells were washed with phosphate-buffered saline (PBS) to remove the quantum dots that were not captured by the cells.

For the cell uptake assay, different concentrations were used: (i) the same concentration in which all Qdots did not show cytotoxicity in NIH/3T3 cells, that is, 20 µg·mL^−1^ and (ii) the maximum concentration in which each quantum dot separately showed no loss of cell viability in NIH/3T3 cells, being 20 μg·mL^−1^ for ZnOGd Qdots, 50 μg·mL^−1^ for the ZnOGd-GCIS/ZnS Qdots and, for GCIS/ZnS Qdots, two different concentrations: 50 μg·mL^−1^ and 250 μg·mL^−1^. Figure 8 shows the results obtained using an excitation wavelength of 360 nm and an emission wavelength of 550 nm. When comparing the three Qdots at 20 μg·mL^−1^ concentration, a greater cellular uptake was observed for the conjugated Qdots (ZnOGd-GCIS/ZnS 75-25). However, when the concentration was increased to 50 μg·mL^−1^, there was a small increase in cell uptake. Regardless of the concentration tested, the conjugated Qdots showed greater cell uptake. Furthermore, a 24-h incubation did not significantly increase the uptake of these Qdots in NIH/3T3 cells. On the other hand, it is important to highlight that no decrease in the photoluminescence intensity was observed after 24 h of incubation, which means that the labelling was stable for at least 24 h in contact with the cells.

## 4. Conclusions

The association of ZnOGd with GCIS/ZnS Qdots by heat treatment showed good photoluminescence properties. Infrared spectroscopy, XRD and TG analyses showed that ZnOGd and GCIS/ZnS Qdots interact with each other, forming a new structure. The conjugated Qdots can be excited using different lamps, not only in the UV range that can cause tissue damage. The conjugated Qdots showed an intermediate PL spectrum. There was a PL redshift for the conjugated Qdots compared with ZnOGd Qdots, even when small proportions of GCIS/ZnS were used, which constituted a more favorable region to obtain biological images. Thus, a new, interesting material was obtained with optimized photoluminescence properties. Furthermore, ZnOGd-GCIS/ZnS Qdots were modified with GPTMS and HTMS to be dispersed in water and an oil phase, respectively. The HTMS-Qdots were associated with LNC formulation. In both cases, we had excellent T2 contrast in magnetic resonance images. Cytotoxicity assay showed an ideal in vitro concentration range for GCIS/ZnS Qdots (<250 μg·mL^−1^), with appropriate biocompatibility. The new Qdots developed (ZnOGd-GCIS/ZnS), mostly constituted of ZnOGd (75%), had less cytotoxicity when compared to ZnOGd, as well as greater cell uptake. Thus, new Qdots were developed and it was possible to obtain both magnetic resonance and luminescence images at the same time. These techniques are complementary and the development of a single nanostructure with optimized properties overcomes their respective limitations.

## Figures and Tables

**Table 1:** Quantum dots (Qdots) proportions used for the conjugation step.

ZnOGd (%)	GCIS/ZnS (%)
98	2
90	10
75	25
50	50

**Table 2:** Photoluminescence (PL) spectra of Qdot powders.

Sample	PL (nm) λ_exc_: 360 nm	PL (nm) λ_exc_: 500 nm
ZnOGd	550	N.D
GCIS/ZnS	620	620
98-2	576	N.D
90-10	580	593
75-25	603	613
50-50	618	620

## Data Availability

The data presented in this study are available on request from the corresponding author.
